# The Dispersion of Health Information–Seeking Behavior and Health Literacy in a State in the Southern United States: Cross-sectional Study

**DOI:** 10.2196/34708

**Published:** 2022-06-15

**Authors:** Nikita Rao, Elizabeth L Tighe, Iris Feinberg

**Affiliations:** 1 Mark Chaffin Center for Healthy Development School of Public Health Georgia State University Atlanta, GA United States; 2 Deparment of Psychology Georgia State University Atlanta, GA United States; 3 Adult Literacy Research Center Department of Learning Sciences Georgia State University Atlanta, GA United States

**Keywords:** health information–seeking behavior, health literacy, digital literacy, information retrieval, health literacy questionnaire

## Abstract

**Background:**

The transmission of health information from in-person communication to web-based sources has changed over time. Patients can find, understand, and use their health information without meeting a health care provider and are able to participate more in their health care management. In recent years, the internet has emerged as the primary source of health information, although clinical providers remain the most credible source. The ease of access, anonymity, and busy schedules may be motivating factors to seek health information on the web. Social media has surfaced as a popular source of health information, as it can provide news in real time. The increase in the breadth and depth of health information available on the web has also led to a plethora of misinformation, and individuals are often unable to discern facts from fiction. Competencies in health literacy (HL) can help individuals better understand health information and enhance patient decision-making, as adequate HL is a precursor to positive health information–seeking behaviors (HISBs). Several factors such as age, sex, and socioeconomic status are known to moderate the association between HL and HISBs.

**Objective:**

In this study, we aimed to examine the relationship between HL and HISBs in individuals living in a southern state in the United States by considering different demographic factors.

**Methods:**

Participants aged ≥18 years were recruited using Qualtrics Research Services and stratified to match the statewide demographic characteristics of race and age. Demographics and source and frequency of health information were collected. The Health Literacy Questionnaire was used to collect self-reported HL experiences. SPSS (version 27; IBM Corp) was used for the analysis.

**Results:**

A total of 520 participants met the criteria and completed the survey (mean age 36.3, SD 12.79 years). The internet was cited as the most used source of health information (mean 2.41, SD 0.93). Females are more likely to seek health information from physicians than males (*r*=0.121; *P*=.006). Older individuals are less likely to seek health information from the internet (*r*=−0.108; *P*=.02), social media (*r*=−0.225; *P*<.001), and friends (*r*=−0.090; *P*=.045) than younger individuals. Cluster analysis demonstrated that individuals with higher levels of HISBs were more likely to seek information from multiple sources than those with lower levels of HISBs (mean range 3.05-4.09, SD range 0.57-0.66; *P*<.001).

**Conclusions:**

Age and sex are significantly associated with HISB. Older adults may benefit from web-based resources to monitor their health conditions. Higher levels of HL are significantly associated with greater HISB. Targeted strategies to improve HISB among individuals with lower levels of HL may improve their access, understanding, and use of health information.

## Introduction

### Background

Health information–seeking behavior (HISB) is a complex construct that refers to the ways in which individuals seek information about health, illnesses, and health choices [1]. Armed with more knowledge, patients can participate more in their health care management, which has been shown to improve the effectiveness and efficiency of health care services and clinical outcomes [2,3]. Traditionally, health information is primarily communicated directly by health care providers to patients. Over time, information dissemination has transitioned from solely patient-provider interactions to obtaining information through Google searches, web-based communities, and one’s social groups [4]. The predictors of HISB can be both contextual and personal, such as the environment, sociodemographic variables, or internal beliefs [5]. The advent of technologies including the internet, smartphones, and social media has prompted a drastic change in how people seek and access health information. Understanding how people seek health information in the digital age is a critical step in developing optimal health information delivery.

While digital health information provides a new landscape for HISB, clinical providers remain one of the most credible sources of health information, especially when it concerns major illnesses [6,7]. This is true irrespective of age or sex [6,7]. The relationship between the patient and provider is mediated by trust, which could be influenced by a patient’s past experiences with their physicians and their perception of quality of care [8]. In addition, those with health insurance are more likely to communicate in-person with health care providers than those who are uninsured; one cause may be the inability to afford visits owing to high out-of-pocket expenses [9]. While clinical providers remain at the forefront of trustworthiness, the internet has emerged as the most frequently used source of health information [8]. Younger people, those with higher education, those with health insurance, being female, and people of color are more likely to use the internet to obtain health information than older people, those with lower education, the uninsured, being male, and White people [9,10]. Ease of access, anonymity, and busy schedules may be motivating factors to seek health information on the internet [10].

Health information from the internet also functions to supplement or cross-reference health information obtained elsewhere, such as from providers, especially in older adults as compared with younger adults [11]. Individuals also rely on the internet to stay abreast of new information in real time, with social media being one of the primary sources to stay informed in this manner. Social media use is most prevalent among millennials (those born between 1980 and 1995) and Gen Z (those born between 1996 and 2012) as a conduit of social support, communication, and obtaining and sharing all kinds of information, including health information [12,13]. The demographics of users on social media are similar to other web-based HISBs, in that being female, younger people, having higher levels of education, and people of color are using social media more than being male, older people, having lower levels of education, and White people [12,13]. In addition, social media’s use of images, videos, and infographics provides information in a comprehensible and visually appealing manner [12]. Instagram, Snapchat, Facebook, and YouTube are popular platforms for seeking health information [12].

### Health Information Seeking

While the younger generations are attuned to seeking health information on the web, the older generation maintains some habit of obtaining health information through traditionally printed materials such as books, newspapers, and magazines [13,14]. People who are older, have lower socioeconomic status, and have low digital literacy may rely on traditional media (printed materials, radio, or television) for health information [14]. Some studies show that non-English speakers were found to favor printed materials, which may be due to their availability in multiple languages [15]. This can pose a problem as health care providers are transitioning to web-based systems to disperse health information, rendering print sources as an antiquated form of health information dissemination [16]. Furthermore, research also shows that individuals with acute and episodic illnesses may also actively seek health information from friends and family [17]. However, when requiring information that is more specialized, such as with serious conditions such as cancer, stroke, and heart disease, friends and family are less frequently consulted [18]. Studies have shown mixed results with friends and family as both a frequent and infrequent source of health information [17,19]. Although friends and family may influence decision-making and provide social support, their role in HISB remains inconsistent.

The numerous methods of acquiring health information have also led to an increase in the breadth of information available. Both accurate and inaccurate information coexist, and there is no overarching regulation to ensure the validity or reliability of information. A study assessing the validity of the search terms “vaccine safety” and “vaccine danger” found that 55% of search results contained inaccurate information within the first 2 pages [19]. Another study on the accuracy of reproductive health information on the internet revealed that it took an average of 4 searches on a search engine to find relevant topics within the mass of media available on the web, indicating an inefficiency in finding health information (FHI) [20]. The wealth of social media platforms has only exacerbated this inefficiency. Although social media is a powerful medium for health information, individuals are now faced with information overload, uncertainty about the validity of their findings, misinformation, disinformation, and often conflicting information [21]. In this manner, information overload can cause stress and confusion for those experiencing it and can reduce their accuracy in health-related decision-making [21].

In the context of the COVID-19 pandemic, these stressors in HISB have been exacerbated by an onslaught of new and ever-evolving information from multiple scientific and nonscientific sources. The global scale and frequent evolution of COVID-19 has led to an infodemic or an overabundance of information [22]. The breadth and spread of misinformation within this context can sometimes lead to dangerous health consequences as misinformation is circulated and absorbed at a faster rate than accurate information [22], changing both people’s understanding of COVID-19 as well as their potentially risky behaviors [23]. A lack of understanding of information related to COVID-19 has been associated with numerous negative health outcomes, including poor mental health, unwillingness to be vaccinated, and discontinuation of healthy preventive behaviors [24].

Although digital media often becomes the primary source of information during global health crises, the availability of many platforms and people’s differential access and abilities in using these media are important factors in developing successful communication strategies to mitigate the risk of misinformation [25]. Digital health equity, including access to digital health information and language barriers, should also be considered when analyzing HISB [26]. Vulnerable populations that may not know how to access this information may require special attention and communication [25,26], as challenges associated with an individual’s low health literacy (HL) and structural inequities can be exacerbated [27]. The proliferation and communication of health information during the pandemic has demonstrated the importance of the concept of HL by Nutbeam [28] as an important aspect in successfully combating information overload and correctly using health information [29].

### Health Literacy and Health Information–Seeking Behaviors

The concept of HL has broadened over time, from a definition of understanding words and numbers in a medical context to the communication, understanding, and use of health knowledge in an interconnected manner [30]. Researchers agree that people should possess several competencies to find, understand, and use health information, including verbal, oral, decision-making, and numeracy skills [28]. Nutbeam [28] categorizes HL into 3 contexts: functional, interactive, and critical. Functional literacy is the ability to read and write, interactive literacy involves the application of health information to everyday circumstances, and critical literacy skills allow for the use of this information to exert control over life events [31]. Nutbeam [28] affirms the importance of distinguishing between these types of literacy and their practical applications. This ternary model of HL is used in several HL studies to map the development of HL competencies beyond the accumulation of basic health knowledge. This model has been used to develop competencies in a wide range of HL studies, including nutrition, patient decision-making, and children’s HL practices [32-34]. Chin et al [35] promote a model of HL that encompasses processing capacity (working memory), general knowledge (ie, vocabulary), and specific health knowledge. This model, while structured differently than the model by Nutbeam [28], also emphasizes how these contexts are central to the overall accumulation of HL. Manganello [36] has expanded upon the model by Nutbeam [28] to include *media literacy*, which is defined as the ability to critically evaluate media messages. Zarcadoolas et al [37] also affirmed the importance of multilevel domains in understanding HL. While their model includes the constructs of *fundamental literacy, civic literacy, science literacy,* and *cultural literacy*, they posit that developing the public’s HL needs to come from a multifaceted perspective [37].

With the rapid development of digital media, eHealth literacy has emerged as the use of information and communication technology to improve access to health care and health information [38]. eHealth literacy expands upon the traditional concept of HL and is associated with similar variables, such as age, education, income, culture, and experience using digital media [39]. Several studies and reviews have found that there is great potential for eHealth and eHealth tools as a manner of patient care and communication [38,40-42]. However, several challenges hinder its efficiency, including the type of technology used, the social environment, its evolving definition and measurements, and a lack of theoretical grounding in developing interventions [40]. In addition, the concept of eHealth literacy is significantly associated with an individual’s level of overall HL, with higher overall HL positively correlated with greater eHealth literacy [38]. As such, while eHealth literacy is a topical and dynamic field, it is important to first evaluate the overall HL in a population as an antecedent to eHealth literacy.

The Health Literacy Questionnaire (HLQ) measures multidimensional, psychometric aspects of HL. By more than simply measuring whether an individual can read and write, the HLQ measures people’s lived experiences of HL using self-reported experiences [43]. The HLQ comprises 9 scales that report patient-centered outcomes so that practitioners may be able to improve their interventions [43]. Studies in different samples from different populations have shown the HLQ to be an appropriate and strong measure of HL from a patient perspective [44-46], demonstrating strong internal consistency. Furthermore, the HLQ has been proven to allow for clinicians to better understand a patient’s HL from their perspective and enable better communication and engagement between the patient and provider, leading to better health outcomes [46]. In addition, the HLQ scales can be organized into 3 categories in the Nutbeam model of HL [43]. Categorizing the HLQ scales into organizational schema in the Nutbeam model highlights how interventions for a specific domain under a scale can influence a broader concept of HL.

Of great importance in how people obtain and use information is how organizations provide health information. Organizational HL (OHL) plays an important role in information acquisition. OHL is the extent to which an organization’s provision of health information is at a level where individuals can read, understand, and use it to make decisions about their health [47]. Successful OHL practices can lead to increased perceived quality and satisfaction of care for patients, ultimately leading to better clinical health outcomes [47,48]. Several guidelines provide evidence-based recommendations for OHL. Many interventions attempt to measure and develop individual HL, but it is of equal or greater significance that organizations include and measure HL in their overall planning for quality of care [49]. Lower levels of HL may deter patient participation in care and can worsen the relationship between the patient and provider [48]. The ultimate purpose of this patient-centered approach is to support patients with their navigation of the health care system and access to, understanding of, and use of health information.

HL and HISB are inextricably linked, as the ability to seek, use, and comprehend health information requires a certain level of HL. In this way, adequate HL is a precursor to positive HISB [50]. Although significant correlations have been found between higher levels of HL and increased HISB, studies suggest that the link between HISB and HL is moderated by other factors, such as social networks, socioeconomic factors, and motivation to seek information [51]. Knowledge transfer within families and communities, social capital, and social engagement in the community contribute to an individual’s level of both HL and HISB [40]. Age and sex are well-known factors that affect HL and HISB. Younger people and being female tend to have higher levels of HL, and their HISB differs significantly from those of older people and being male, respectively [13,16]. Being female and younger people are likely to find information more easily as compared with being male and older people [52]. HL has also been shown to decline with age and can lead to negative health outcomes, including increased mortality [53], although being female was associated with higher HL than being male in older age [40]. Individuals with higher levels of education are more likely to seek health information from a variety of sources [18] whereas those with lower levels of HL are less likely to do so [18]. In addition, those with lower levels of HL are more likely to need multiple sources of health information to be able to digest health knowledge and apply it [54].

Some studies show that individual characteristics, such as lower levels of income and lack of access to care associated with low HL are generally concentrated in rural areas, whereas those with higher levels of HL tend to reside in more urban areas [52]. However, these differences between urban and rural HL levels tend to disappear once factors such as age, sex, education, and income are accounted for [55]. As people in urban areas can also have low levels of HL, social and financial capital may be more strongly associated with HISB than HL itself [56]. Possible risk factors that may contribute to differences in urban and rural HL levels could be lack of employment, male sex, and language [57]. However, no single factor has been identified as significantly correlated with HL, and the differences between urban and rural levels of HL remain misunderstood. Significant associations have been found between urbanicity and HL and subsequent health outcomes, as those with higher levels of HL in urban areas are more likely to adopt healthy lifestyles [58]. Those characteristics of urbanicity associated with lower levels of HL are also correlated with HISB [59]. Urbanicity is also found to interact with HL to predict HISB. Chen et al [59] found that among rural residents, limited HL was associated with lower odds of access to health information, while among urban residents, HL was not associated with access to health information.

### Study Context

Health disparities are exacerbated by macrosocial and microsocial factors, such as lack of health care access, low reading skills, health care costs, and geography. People living in the rural southern United States have higher rates of morbidity and mortality compared with their urban counterparts and those in other rural areas, with people of color experiencing higher rates of death and disease as compared with their White counterparts [60]. Most of these states have low rates of health care insurance, even with the expansion of Medicaid under the Affordable Care Act, leading to poorer health outcomes. In states that have chosen not to expand Medicaid, Texas, Georgia, and Florida account for half of the uninsured population [61]. Our study focused on a sample in Georgia, which ranks poorly for a variety of factors that influence HISB, including socioeconomic factors, health behaviors, and chronic disease [62]. Low-income and ethnic minorities are more likely to have poorer health outcomes such as heart conditions, cancer, stroke, and obesity than their White counterparts with higher income, exacerbating their negative health outcomes as they are unable to access health information in the traditional sense [62,63]. Understanding the structural and individual factors that influence health disparities in Georgia will allow us to understand the variables that influence HISB in this population as an essential first step in developing effective and accessible health information.

### Goal of This Study

In this study, we examined the relationship between HL and HISB considering different demographic factors, such as age, sex, highest level of educational attainment, health insurance status, and county type as rural or urban. The HLQ was used to examine the lived HL experiences of individuals in a southern US state in terms of understanding, accessing, and using health information and health services as a measure of patient-reported outcomes. The purpose of this study was to examine HL and HISBs among a representative sample of adults in a southern US state by answering the following research questions:

- What are the average scores on the HISB scales?
- Are demographics (sex, age, education level, and county) related to the HISB scales?
- Are HISB scales predictive of HL outcomes (have sufficient information [HSI], critical appraisal [CA], FHI, and understanding health information [UHI])?
- Are there distinct clusters of participants based on HISB responses? Do these clusters differ by HL outcomes and participant demographics?

## Methods

### Recruitment

Participants who lived in Georgia and were aged ≥18 years were recruited using Qualtrics Research Services and stratified to match statewide demographic characteristics of geography and race (Explore Census Data [64]). Recruitment was conducted through email invitations or prompt survey platform prompts. Participants had individual incentive agreements with Qualtrics Research Services that included cash, gift cards, or retail store miles.

### Measures

Demographic information on age, sex, race, highest level of educational attainment, health insurance status, and zip code was also collected. We assessed the frequency of sources of health information (from a lot to none) for printed materials, the internet, social media, physicians, and family and friends. We used the HLQ to collect different aspects of lived HL experiences related to accessing, understanding, and using health information: HSI, CA of health information, FHI, and UHI. All scales contain 4 to 6 items scored on a Likert-type scale; HSI and CA scales 1 to 4 have four response options (strongly disagree, disagree, agree, and strongly agree), and FHI and UHI scales 5 to 9 have five response options (cannot do, very difficult, quite difficult, easy, and very easy).

### Statistical Analysis

We used SPSS (version 27; IBM Corp, 2020) for the analysis. Descriptive statistics included means, SDs, frequencies, and chi-square calculations. A 2-step cluster analysis was performed using the 4 HL scales.

### Ethics Approval

The study was approved by the institutional review board of Georgia State University under the approval number H21522.

## Results

### Recruitment

Out of those who responded to the survey, 57.4% (520/905) met all the criteria and completed the survey. Table 1 presents the demographic characteristics of the participants. The mean age was 36.3 (SD 12.79; range 18-80) years. Racial categories matched state stratification rates. Educational attainment was split evenly between less than high school diploma, some college education, and college degree. Health insurance status showed that 72.6% (378/520) of the participants answered yes. Out of 520 respondents, 264 (50.8%) were urban county dwellers, which is similar to state demographics.

**Table 1:** Demographic data for overall sample (n=520).

Variable		Participants, n (%)
Female		371 (71.2)
**Race**		
	Black or African American	167 (32.1)
	White	301 (58)
	Asian	28 (5.4)
	Hispanic	13 (2.5)
	Other	12 (2)
**Educational attainment**		
	High school diploma or less	160 (30.7)
	Some college education	177 (34)
	College degree	184 (35.3)
Has health insurance		378 (72.6)
**Geography**		
	Urban county	264 (50.8)
	Rural county	256 (49.2)

### Statistical Analysis

To address the first research question, “What are the average scores on the 5 health information–seeking behavior (HISB) scales?” means, SDs, and range values of the HISB scales are presented in Table 2. The scores ranged from 1 (none) to 4 (a lot).

To address the second research question, “Are demographics (sex, age, educational level, and county) related to the HISB scales?” Spearman rank correlations between demographics (sex, age, educational level, and county) and the 5 HISB scales are shown in Table 3. Of note, sex was significantly associated with the physician HISB scale (*r_s_*=0.121; *P*=.01), such that being female was more likely to be associated with seeking health information from physicians. Age was significantly negatively associated with the internet (*r*=−0.108; *P*=.02), social media (*r*=−0.225; *P*<.001), and family and friends (*r*=−0.090; *P*=.045) HISB scales. This indicates that as age increases, individuals are less likely to seek health information from the internet, social media, and family and friends. There were no significant associations between educational level and county with any of the HISB scales (county with printed materials: *r*=−0.051, *P*=.25; county with IR: *r*=−0.062, *P*=.16; county with social media: *r*=0.010, *P*=.83; county with doctor or looks like physicians: *r*=−0.048, *P*=.28; county with family and friends: *r*=0.053, *P*=.23; education with printed materials *r*=−0.007, *P*=.88; education with IR: *r*=.033, *P*=.46; education with social media: *r*=0.057, *P*=.19; education with doctor or looks like physicians: *r*=0.045, *P*=.31; education with family and friends *r*=0.023, *P*=.61). As expected, the 5 HISB scales were all positively and significantly related printed materials with IR: *r*=0.100, *P*=.02; printed materials with social media: *r*=0.331, *P*<.001; printed materials with doctor or looks like physicians: *r*=0.118, *P*=.007; printed materials with family and friends: *r*=0.221, *P*<.001; IR with social media: *r*=0.296, *P*<.001; IR with doctor or looks like physicians: r=0.203, *P*<.001; IR with family and friends: *r*=0.122, *P*=.005; social media with doctor or looks like physicians: *r*=0.095, *P*=.03; social media with family and friends: *r*=0.330, *P*<.001; doctor or looks like physicians with family and friends: *r*=0.274, *P*<.001).

To address the third research question, “Are HISB scales predictive of HL outcomes (HSI, CA, FHI, and UHI)?” a series of multiple regression analyses were conducted using SPSS. We ran 4 regression analyses, which included all HISB scales predicting each HL outcome (HSI, CA, FHI, and UHI; Tables 4-7). Before running the regression analyses, we dummy coded the 5 HISB scales. For each scale, 2 dummy codes were created and the “a lot” response served as the reference group: none or little (labeled D1) and some (labeled D2). We chose to dummy code the variables as the scale was ordinal and only ranged from 1 (none) to 4 (a lot). We considered these ordinal variables as categorical variables, which requires dummy coding. We collapsed the “none” and “little” groups into 1 group owing to low responses in these categories. As we were most interested in the comparison with the “a lot” group, we used it as the reference. These analyses helped us determine which HISB scales were most important for each HL outcome. For all 4 regression analyses, at least one dummy code for printed materials, internet, and physician HISB scales was uniquely predictive of all HL outcomes (see Tables 3-6 for estimates and unique *R*^2^ values). Social media and family and friends HISB scales were not uniquely predictive of any of our HL outcomes. The HISB scales accounted for 0.223 to 0.331 of the variances in our 4 HL outcomes.

**Figure 1:**
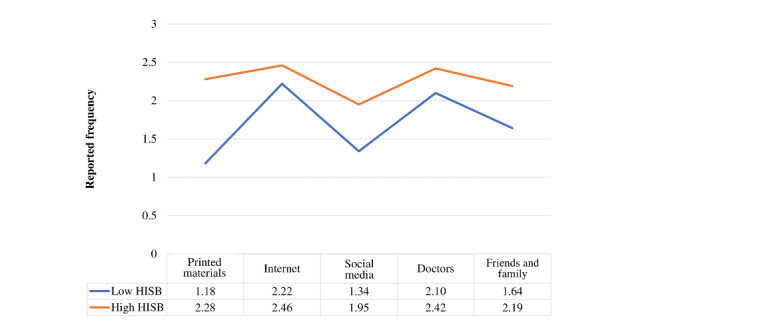
Two clusters based on 5 health information–seeking behaviors (HISBs).

To address the fourth research question, “Are there distinct clusters of participants based on HISB responses? Do these clusters differ by HL outcomes and participant demographics?” we conducted a 2-step cluster analysis using SPSS with the 5 HISB scales (all scored 1-4). The results indicated that there were 2 distinct HISB clusters based on the 5 scales (Figure 1). We have descriptively labeled the clusters as “high HISB” (203/520, 39%) and “low HISB” (317/520, 60.9%). The pattern of responses to the HISB scales was similar across the 2 clusters; however, the high HISB cluster had more uniform and stable responses, with the exception of social media, which had the lowest reported health-seeking behavior (mean 1.95, SD 0.75). The low HISB cluster had printed materials followed by social media as their least frequent sources of health information (means 1.18 and 1.34, respectively, SD 0.38 and 0.58 respectively; Figure 1). We used chi-square difference tests to examine whether the clusters were differentiated based on 3 categorical demographics (sex, educational level, and county), and no significant differences were found (county, *P*=.87; education, *P*=.25; sex, *P*=.96). We used a 2-tailed *t* test to examine whether the clusters differed based on age, and there was no significant difference (*P=*.33). Finally, we conducted a series of 2-tailed *t* tests to examine whether the clusters differed based on our HL scales (HSI, CA, FHI, UHI, and actively managing health). There were significant mean differences for all 5 HL scales, such that the high HISB cluster had significantly higher means across all scales (means 3.05-4.09, SD 0.57-0.66; *P*<.001) than the low HISB cluster (means 2.63-3.78, SD 0.52-0.75; *P*<.001).

**Table 2:** Descriptives of health information–seeking behavior (HISB).

Variable	Value, mean (SD; range)
HISB printed materials	2.41 (0.93; 1-4)
HISB internet	3.28 (0.81; 1-4)
HISB social media	2.31 (1.00; 1-4)
HISB physicians	3.19 (0.82; 1-4)
HISB family and friends	2.78 (0.82; 1-4)

**Table 3:** Spearman rank correlations among the health information–seeking behavior (HISB) scales and demographics^a^.

Variable		Sex	Age (years)	Education level	County	HISB print material	HISB internet	HISB social media	HISB physicians	HISB family and friends
**Sex**										
	*r*	1	−0.038	0.018	0.081	−0.025	0.075	0.019	0.121	0.060
	*P* value	—^b^	.40	.69	.07	.57	.09	.67	.006	.18
**Age**										
	*r*	—	1	0.051	−0.028	−0.048	−0.108	−0.225	0.053	−0.090
	*P* value	—	—	.25	.54	.29	.02	<.001	.24	.045
**Education level**										
	*r*	—	—	1	−0.085	−0.007	0.033	0.057	0.045	0.023
	*P* value	—	—	—	.05	.88	.46	.19	.31	.61
**County**										
	*r*	—	—	—	1	−0.051	−0.062	0.010	−0.048	0.053
	*P* value	—	—	—	—	.25	.16	.83	.28	.23
**HISB print materials**										
	*r*	—	—	—	—	1	0.100	0.331	0.118	0.221
	*P* value	—	—	—	—	—	.02	<.001	.007	<.001
**HISB internet**										
	*r*	—	—	—	—	—	1	0.296	0.203	0.122
	*P* value	—	—	—	—	—	—	<.001	<.001	.005
**HISB social media**										
	*r*	—	—	—	—	—	—	1	0.095	0.330
	*P* value	—	—	—	—	—	—	—	.03	<.001
**HISB physicians**										
	*r*	—	—	—	—	—	—	—	1	0.274
	*P* value	—	—	—	—	—	—	—	—	<.001
**HISB** **family and friends**										
	*r*	—	—	—	—	—	—	—	—	1
	*P* value	—	—	—	—	—	—	—	—	—

^a^The sample size ranges from 497 to 520. To interpret the direction of the correlations for dichotomous demographic variables, being female, some college or more, and rural county were all coded higher.

^b^Not applicable.

**Table 4:** Health information–seeking behavior scales predicting have sufficient information^a^.

Predictor	Unique *R*^2^	Coefficient (SE)	*t* test (*df*)	*P* value
Printed materials_D1^b^	0.056	−0.423 (0.070)	6.02 (508)	<.001
Printed materials_D2^c^	0.046	−0.381 (0.069)	5.50 (508)	<.001
Internet_D1	0.013	−0.130 (0.045)	2.89 (508)	.004
Internet_D2	N/A^d^	−0.078 (0.044)	1.77 (508)	.08
Social media_D1	N/A	−0.047 (0.068)	0.69 (508)	.49
Social media_D2	N/A	−0.029 (0.065)	0.44 (508)	.66
Doctor_D1	0.064	−0.294 (0.045)	6.47 (508)	<.001
Doctor_D2	0.011	−0.118 (0.044)	2.68 (508)	.008
Family and friends_D1	N/A	−0.056 (0.060)	0.93 (508)	.36
Family and friends_D2	N/A	−0.037 (0.058)	0.64 (508)	.52

^a^These are standardized coefficients. Total *R^2^*=0.223. The response “a lot” served as the reference group for all dummy codes.

^b^D1: dummy code representing “none or little.”

^c^D2: dummy code representing “some.”

^d^N/A: not applicable.

**Table 5:** Health information–seeking behavior scales predicting critical appraisal^a^.

Predictor	Unique *R*^2^	Coefficient (SE)	*t* test (*df*)	*P* value
Printed material_D1^b^	0.072	−0.488 (0.067)	7.31 (508)	<.001
Printed material_D2^c^	0.037	−0.344 (0.066)	5.23 (508)	<.001
Internet_D1	0.043	−0.239 (0.042)	5.65 (508)	<.001
Internet_D2	0.019	−0.156 (0.042)	3.74 (508)	<.001
Social media_D1	N/A^d^	0.012 (0.064)	0.19 (508)	.85
Social media_D2	N/A	0.033 (0.061)	0.537 (508)	.59
Doctor_D1	0.070	−0.307 (0.043)	7.18 (508)	<.001
Doctor_D2	0.014	−0.135 (0.041)	3.25 (508)	<.001
Family and friends_D1	N/A	−0.057 (0.056)	1.00 (508)	.32
Family and friends_D2	N/A	−0.015 (0.055)	0.27 (508)	.79

^a^These are standardized coefficients. Total *R^2^*=0.312. The response “a lot” served as the reference group for all dummy codes.

^b^D1: dummy code representing “none or little.”

^c^D2: dummy code representing “some.”

^d^N/A: not applicable.

**Table 6:** Health information–seeking behavior scales predicting finding health information^a^.

Predictor	Unique *R*^2^	Coefficient (SE)	*t* test (*df*)	*P* value
Printed material_D1^b^	0.018	−0.238 (0.068)	3.48 (509)	<.001
Printed material_D2^c^	N/A^d^	−0.125 (0.067)	1.85 (509)	.06
Internet_D1	0.022	−0.169 (0.044)	3.87 (509)	<.001
Internet_D2	N/A	−0.067 (0.043)	1.56 (509)	.12
Social media_D1	N/A	0.117 (0.066)	1.77 (509)	.08
Social media_D2	N/A	−0.106 (0.063)	1.68 (509)	.09
Doctor_D1	0.099	−0.365 (0.044)	8.26 (509)	<.001
Doctor_D2	0.057	−0.269 (0.043)	6.27 (509)	<.001
Family and friends_D1	N/A	−0.044 (0.058)	0.75 (509)	.45
Family and friends_D2	N/A	−0.038 (0.057)	0.68 (509)	.50

^a^These are standardized coefficients. Total *R^2^*=0.263. The response “a lot” served as the reference group for all dummy codes.

^b^D1: dummy code representing “none or little.”

^c^D2: dummy code representing “some.”

^d^N/A: not applicable.

**Table 7:** Health information–seeking behavior scales predicting understanding health information^a^.

Predictor	Unique *R*^2^	Coefficient (SE)	*t* test (*df*)	*P* value
Printed material_D1^b^	0.020	−0.253 (0.069)	3.69 (509)	<.001
Printed material_D2^c^	0.016	−0.225 (0.068)	3.32 (509)	.001
Internet_D1	0.011	−0.121 (0.044)	2.76 (509)	.006
Internet_D2	N/A^d^	−0.031 (0.043)	0.71 (509)	.48
Social media_D1	N/A	−0.086 (0.066)	1.30 (509)	.19
Social media_D2	N/A	−0.086 (0.064)	1.36 (509)	.18
Doctor_D1	0.114	−0.391 (0.044)	8.81 (509)	<.001
Doctor_D2	0.073	−0.304 (0.043)	7.06 (509)	<.001
Family and friends_D1	N/A	−0.036 (0.059)	0.61 (509)	.54
Family and friends_D2	N/A	−0.029 (0.057)	0.51 (509)	.61

^a^These are standardized coefficients. Total *R^2^*=0.256. The response “a lot” served as the reference group for all dummy codes.

^b^D1: dummy code representing “none or little.”

^c^D2: dummy code representing “some.”

^d^N/A: not applicable.

## Discussion

### Principal Findings

Our study highlights several important links between HISB and HL: (1) as age increases, people are less likely to seek health information from the internet and social media; (2) seeking health information from social media is not predictive of HL outcomes and is the least-used source of health information for people with high and low HL levels; and (3) people with high HL consistently exhibit more HISBs across multiple sources than those with low HL.

Although internet use has significantly increased in the past few years, disparities remain owing to age, gender, race, and socioeconomic status, which may persist in the digital gap between generations and among populations [65]. Studies show that people of all ages may prefer more traditional, printed medium or health care professionals for health information yet also seek corroboration, new information, or different perspectives from web-based HISBs [66]. Among those who use the internet, 79% have looked for health information of one kind or another, and 55% of these *online diagnosers* have spoken with a clinician about what they have found on the web [67]. Health information available on the web might be especially important for those with sensitive or stigmatized health issues such as drug use, unplanned pregnancies, and sexually transmitted diseases [68]. Older adults who may not have digital skills or digital access may not be able to access important health information, participate in decision-making with their health providers, reach provider websites to access patient portals, or participate in social support networks [69,70]. In addition, among older adults, those with higher cognitive skills are more likely to seek health information on the web than those with lower cognitive skills [68]. Other studies indicate an increase in internet use in older age groups; however, age was still not considered a predictor of HISB [70]. Although older adults may be heavy users of health services owing to increasing age-related and comorbid illnesses, they tend to be the lowest category of internet and other web-based health service users [71,72]. While these findings are specific to adults in Georgia, they are similar to other findings across the United States and the globe.

Social media allows users to quickly create and share content and participate in broad information sharing and consumption; different theoretical models propose that individuals are looking for action-oriented information, assessment of risk perception and responses, and more broadly, general information gathering [73]. In our study, the use of social media was not predictive of HL outcomes such as HSI, being able to critically appraise health information, FHI, or UHI. In addition, social media was the least used source of health information for individuals with both high and low HL in Georgia. Thus, although social media is a widely used platform for information dissemination, we found that it is not a significant source of health information nor does it appear to be related to HL outcomes. Some studies on information seeking of COVID-19 information, indicate that social media exposure may result in a significant overload of information that could lead to information anxiety and avoidance, thus having a negative impact on both HISB and HL outcomes [74]. Although this study was conducted during the pandemic, it did not focus on COVID-19 health information; rather, questions were asked about general HISBs.

Using cluster analysis, we were able to ascertain a high HISB and a low HISB cluster (39% and 61% of the sample, respectively). The high HISB cluster used all 5 sources of health information significantly more than the low HISB cluster in all HISB categories, and social media was used the least by both clusters. The high and low clusters were not differentiated by sex, educational level, county, or age. Interestingly, both clusters used social media the least as a health information source. Wang et al [75] posit that although social media networks are widely used and may facilitate HISB, they are also the perfect environment for spreading rumors and accurate information, and it is difficult for social media users to ascertain between the two. The lack of control over who can post information on the web has placed additional difficulty on discerning accurate scientific data from misinformation [20]. Social media content also changes quickly; users’ cognitive limits may be maximized, which can lead to information overload, vulnerability, uncertainty, and self-isolation [76]. Therefore, individuals may avoid social media channels when searching for health information.

The high HISB cluster exhibited higher HL across all 4 scales (HSI, CA of health information, FHI, and UHI). This is consistent with prior studies that indicate that having higher HL may influence a preference for information seeking over and above demographic variables [77,78]. This may not be causal; that is, individuals who seek health information may improve their HL owing to motivation for or better access to information in the same way that individuals with higher HL are more confident in seeking health information [77-79]. Studies indicate that patients who have higher HL may also have better patient engagement, have high self-advocacy, participate more in shared decision-making, and have better health outcomes [80-82]. Those patients with low HL may not have the capacity to seek health information from multiple sources because of their lower socioeconomic status, language barriers, and educational differences. Often, as compared with those with higher HL, those with lower HL are more likely to rely on health care providers’ recommendations for their clinical course of action without seeking further information, signifying overlap between HISB and HL in socio-cognitive predictors such as perceived self-efficacy to obtain health information. In these cases, strategies should be implemented to increase patients’ motivation to be informed on how to access, understand, and use health information from other sources. Health education practices targeting these populations may facilitate a greater understanding of clinical information and lead to healthier clinical outcomes [77,83,84].

### Limitations

While this study sample mirrored the demographics of the state, we were only able to reach individuals who have computer access. Thus, we have reported findings only for individuals who have digital access and at least a minimum of digital literacy skills. As the recruitment was performed using web-based channels, sampling bias is a potential limitation of this study, as those who had difficulties in using these channels could be excluded from recruitment. Another limitation is that we were only able to survey participants in 1 southern US state. We stratified the sample to match the statewide demographic characteristics of geography and race but learned after data collection that sex and age distributions are largely skewed. Future studies should construct more complex stratification to account for this skewness in the data. Although we believe the findings are generalizable among Georgia residents, they may not be generalizable across other states.

### Conclusions

Age and sex were significantly associated with HISBs. As older adults are more likely to use health services, they may benefit from having web-based resources to update them on their health status in real time and to provide accessible social support networks. Thus, there is a need to improve HISB skills of and interventions for older adults. Higher levels of HL are associated with greater HISB. Those with lower levels of HL may benefit from targeted strategies to improve their understanding of health information and how to access, understand, and use it, as greater understanding of health information is associated with healthier clinical outcomes. Further studies are needed, specifically those focused on HL, urbanicity, and access to health information.

## Abbreviations

CA: critical appraisal
FHI: finding health information
HISB: health information–seeking behavior
HL: health literacy
HLQ: Health Literacy Questionnaire
HSI: have sufficient information
OHL: organizational health literacy
UHI: understanding health information
